# Deletion of *sc3*, Alone or in Combination with *sc15*, Alters Mycelial Surface Wettability and Substrate-Dependent Adhesion in *Schizophyllum commune*

**DOI:** 10.1021/acs.langmuir.6c04711

**Published:** 2026-10-13

**Authors:** Wenjing Sun, Han A. B. Wösten

**Affiliations:** † Department of Biology, Microbiology, 2717 Utrecht University, Padualaan 8, Utrecht 3548 CH, The Netherlands; ‡ Magan Centre of Applied Mycology, Cranfield University, Cranfield MK43 0AL, U.K.

## Abstract

Fungal mycelium has attracted increasing interest as a sustainable alternative to petroleum-derived adhesives and functional coatings. However, the interfacial mechanisms that govern fungal adhesion and surface interactions are still not fully understood. In this study, we investigated how deletion of the hydrophobin gene *sc3* either alone or together with the hydrophobin-like *sc15* gene impacts extracellular polysaccharide secretion, mycelial surface properties, and adhesion in the basidiomycete *Schizophyllum commune*. Two wild-type strains (H4–8A and 4–39) and the Δ*sc3* and Δ*sc3*Δ*sc15* strains were grown in liquid shaken conditions and under static conditions on glass, Teflon, and wood that had been placed on agar medium. Production of the extracellular polysaccharide schizophyllan was increased in the Δ*sc3* and the Δ*sc3*Δ*sc15* strains when compared to the wild-type strain. Moreover, unlike the wild-type strains, the deletion strains produced a hydrophilic instead of a hydrophobic mycelium at the air-exposed side, which was accompanied by reduced aerial hyphae formation. While all strains adhered strongly to wood, wild-type strains attached more strongly to hydrophobic Teflon than the Δ*sc3* and the Δ*sc3*Δ*sc15* strains. Adhesive bonding strengths on birch wood veneer ranged from 1.31 to 1.83 MPa, with the Δ*sc3*Δ*sc15* mutant showing the highest strength after growth on this substrate under static conditions and Δ*sc3* after growth in liquid shaken cultures. These results demonstrate that deletion of *sc3*, either or not in combination with *sc15* impacts substrate-specific adhesion, while increased formation of schizophyllan in these strains did not consistently correspond to increased bonding.

## Graphical abstract

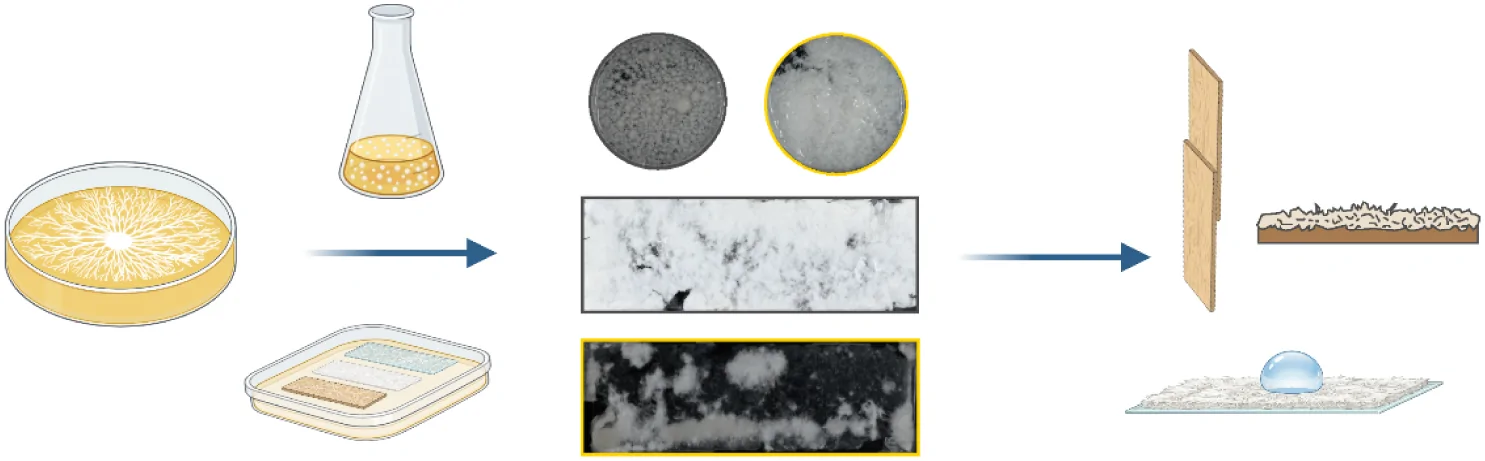

## Introduction

Filamentous fungi produce extensive networks of hyphae that naturally bind and colonize a wide range of substrates.^1,2^ This ability has recently attracted attention in the development of sustainable materials, including mycelium-bound composites, mycelium-based coatings, and adhesives.^3,4^ Compared with conventional petroleum-derived binders, fungal materials offer several advantages, such as production from diverse feedstocks under mild processing conditions and inherent biodegradability. However, the interfacial mechanisms that control fungal adhesion and surface interactions remain insufficiently understood, limiting the rational design of fungal-based bonding systems.

Fungal adhesion arises from a combination of physical and biochemical factors. Hyphal networks can mechanically interlock with porous substrates such as wood, while extracellular polysaccharides and surface proteins contribute to interfacial interactions.^5,6^ Among these components, hydrophobins and hydrophobin-like proteins are of particular interest. Hydrophobins are secreted proteins that are released in the cell wall and/or the culture medium. These proteins have eight conserved cysteine residues that assemble into amphipathic membranes at hydrophilic–hydrophobic interfaces, thereby modifying surface properties.^7–9^ Hydrophobins are known to play key roles in the formation of aerial hyphae, spore dispersal, and surface hydrophobicity and can transform hydrophilic fungal cell walls into highly hydrophobic surfaces, enabling fungi to interact with air, oil, and hydrophobic solid interfaces.^8,9^ Inactivation of the *sc3* hydrophobin gene in the mushroom-forming fungus *Schizophyllum commune* affected the formation of hydrophobic aerial hyphae^10^ as well as adhesion to hydrophobic surfaces.^11^ However, these processes were not completely abolished, explained by the involvement of the SC15 protein as well.^12^ This secreted protein, which is found in the culture medium, the cell wall, and the mucilage that binds hyphae together, is not a hydrophobin as it lacks the conserved cysteine residues. Yet, it contains a hydrophilic N-terminal half and a highly hydrophobic C-terminal half, and therefore, this protein is called a hydrophobin-like protein. Inactivation of the *sc15* gene in the Δ*sc3* background further reduced the formation of hydrophobic aerial hyphae and adherence to hydrophobic surfaces. By contrast, inactivation of *sc15* in the wild-type background did not have any effect on these processes.

Secreted extracellular polysaccharides may also influence fungal adhesion behavior and bonding performance.^6,13^ These polymers influence hydration, viscosity, cohesion, and interfacial behavior and can form hydrated mucilage layers associated with hyphal surfaces.^6,13,14^ Notably, hydrophobin expression has been linked to changes in mucilage production and hyphal wall composition.^15^ Inactivation of the hydrophobin gene *sc3* increased the release of the β-(1–3)-β-(1–6) glucan schizophyllan, while the amount of glucan linked to chitin decreased.^15^

Together, fungal adhesion depends on surface-active proteins and extracellular polysaccharides. At the molecular level, SC3 interacts with schizophyllan and β-(1–3)-glucan, and these interactions can influence its assembly at interfaces.^13,16,17^ Adhesion between hydrophobin layers has also been quantified directly in model interfaces, showing contributions from van der Waals and electrostatic interactions.^18^ However, experimental evidence linking specific molecules to bonding performance remains limited. This study assessed extracellular polysaccharide secretion, surface properties, and adhesion and bonding in the basidiomycete *S. commune*. It is shown that production of schizophyllan was increased in both the Δ*sc3* and the Δ*sc3Δsc15* strains and that the air-exposed side of their mycelium was hydrophilic instead of hydrophobic, like the wild-type. Moreover, attachment and bonding capacity to wood were not affected in the deletion strains, while this was the case for Teflon. These findings provide a basis for understanding how targeted modification of fungal surface-associated proteins can influence adhesion and bonding behavior in biobased material systems.

## Materials and Methods

The overall experimental workflow, including liquid shaken and static cultivation on solid surfaces, surface characterization, attachment testing, and lap-shear bonding, is summarized in Figure 1.

**Figure 1:**
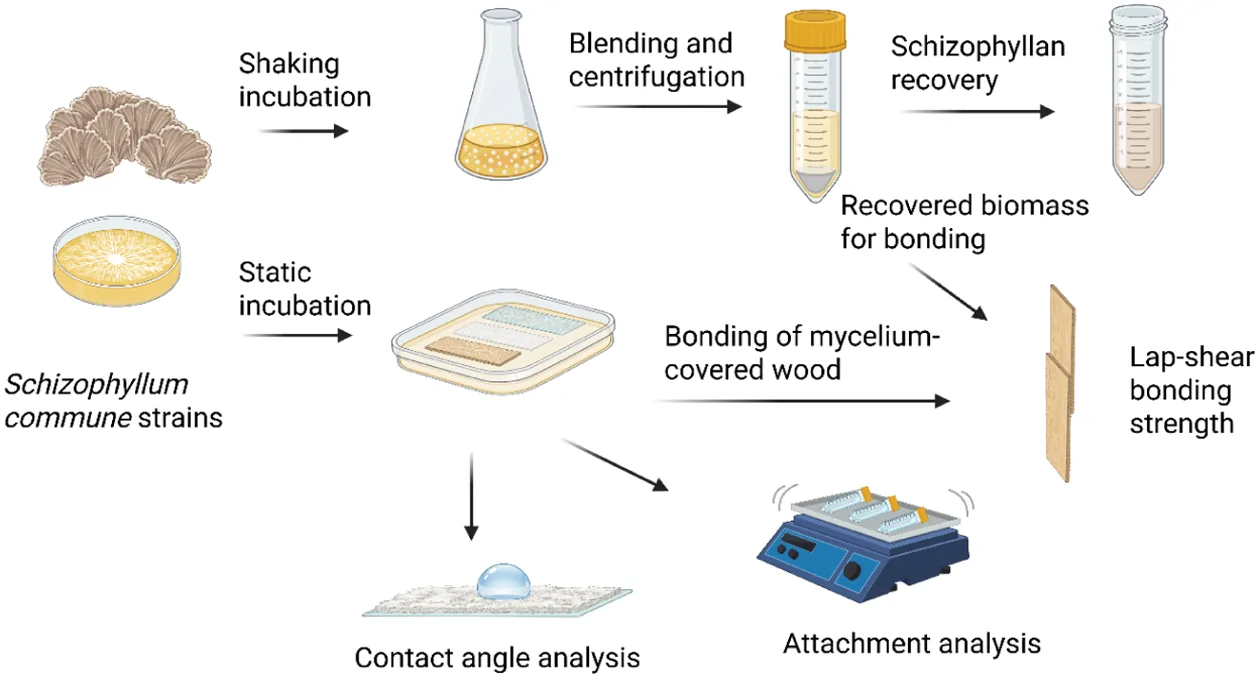
Schematic workflow of the study.

### Strains and Culture Conditions

Wild-type *S. commune* strains H4–8A (matA_43_matB_41_; FGSC no. 9210) and 4–39 (matA_41_matB_41_, FGSC no. 9216) were used in this study, as well as the 4–39-derived deletion strains Δ*sc3* and Δ*sc3*Δ*sc15*.^10,12^ To prepare inoculum, strains were grown for 7 days at 30 °C on minimal medium (MM)^19^ with 1.5% agar (MM-A) reaching a surface area of approximately 20 cm^2^. The mycelium, from which the agar had been removed, was macerated in 50 mL MM for 30 s in a Waring 2-Speed Blender at 18,000 rpm (Waring Laboratory Science, www.waringlab.com). The macerate was mixed with sterilized glycerol (4:1 v/v), and 12.5 mL aliquots were stored at −80 °C until used as an inoculum.

For liquid shaken cultures, 12.5 mL inoculum was added to 37.5 mL MM in a 250 mL Erlenmeyer flask. The culture was incubated at 30 °C in the dark at 200 rpm. After 5 days, the culture was macerated in the Waring 2-Speed Blender (see above), dialyzed (molecular weight cutoff 6,000–8000 Da) 2 times 1 h and 1 time 16 h, and centrifuged at 10,000 g for 10 min. To recover the schizophyllan fraction, the supernatant was mixed with ethanol (2:1, v/v) and stored overnight at 4 °C. The resulting precipitate was washed with 70% ethanol and then dried at 60 °C.

For static growth, 12.5 mL inoculum was spread on square Petri dishes (12 × 12 cm) containing MM-A, on top of which 4 pieces of each substrate were placed: glass slides (76 × 26 × 1 mm), birch veneer (35 × 10 mm with a 0.5–0.7 mm thickness) (Creaknutselen, article number 28290; www.creaknutselen.nl); and polytetrafluoroethylene (PTFE; Teflon, 35 × 10 × 1 mm) (Allesxl, article number ALXNL560993707; www.allesxl.com). Glass slides and Teflon pieces were washed with deionized water and 70% ethanol before autoclaving, while birch veneer pieces were autoclaved before use without pretreatment. Cultures were incubated at 30 °C in the dark for 6 days. Substrates covered with mycelium were carefully removed from the agar plates with a pair of tweezers.

### Scanning Electron Microscopy (SEM)

Surface morphology of mycelium and substrates was examined using a TESCAN VEGA 3 scanning electron microscope. Samples were dried before imaging and mounted on aluminum stubs using conductive carbon tape. Imaging was done with a backscattered electron detector at an accelerating voltage of 10 kV.

### Contact Angle Analysis

Contact angle measurements were carried out at ambient temperature using an optical contact angle measuring and contour analysis system (OCA 15EC, DataPhysics Instruments, www.dataphysics-instruments.com). A 2 μL droplet of deionized water was used for each measurement. A total of 6–8 droplets were placed on 3 biological replicates. Water contact angles of uncolonized glass, Teflon, and wood veneer were also measured using the same procedure.

### Surface Mycelium Attachment on Different Substrates

Surface mycelium attachment was assessed using a stepwise washing detachment assay. Fresh or dried samples of mycelium grown on Teflon and birch veneer were placed individually into 15 mL Eppendorf tubes, while mycelium grown on glass slides were placed in 50 mL Eppendorf tubes. Either 10 mL (for Teflon and wood) or 45 mL (for glass) deionized water was added to each tube, after which the tubes were placed on a shaking platform starting at 50 rpm for 30 min and increasing stepwise by 50 rpm every 30 min up to 350 rpm. The speed at which the mycelium fully detached from the substrate was recorded. Samples (6–7 replicates for each growth condition) that remained attached at 350 rpm were recorded as retained at the maximum tested speed.

### Lap-Shear Bonding Analysis

The adhesion properties of the mycelium from liquid shaken and static incubation conditions were analyzed by producing lapped samples using a hot press. The lapping area of the lap-shear samples was 1 × 1 cm. An amount of 200 g m^–2^ (wet-based) was used for mycelium from liquid shaken cultures. For static-incubation samples, birch veneer pieces carrying directly grown surface mycelium were lapped together with the mycelial surfaces facing each other. The surface-mycelium loading in the static-incubation samples was not normalized, in order to preserve the growth-formed mycelium–wood interface.

The lap-shear samples were hot-pressed for 5 min in a hydraulic press (FONTIJNE LabManual 300, Fontijne Presses, www.fontijnepresses.com) at 120 °C under 1 MPa of pressure. Lap-shear tests were conducted using a mechanical testing machine (LS5, Lloyd Instruments/Ametek, www.ametektest.com) with a 1 kN load cell. Samples were conditioned at 25 °C and 50% RH to a constant mass before heat pressing. The crosshead speed was 0.5 mm min^–1^, and 6–8 replicates were tested for each group.

### Statistical Analysis

Statistical analysis was performed using OriginPro 2025b software (OriginLab Corporation, Northampton, MA, USA). Differences among strains were analyzed by one-way analysis of variance followed by Tukey’s post hoc test (*p* ≤ 0.05). For the stepwise washing detachment assay, individual detachment speeds are shown, while samples retained at 350 rpm are indicated as retained at the maximum tested speed.

## Results and Discussion

### Liquid Shaken Cultures

The two wild-type strains and the Δ*sc3* and the Δ*sc3*Δ*sc15* strains all formed mycelial pellets in liquid shaken cultures (Figure 2A). Biomass formation of the wild-type strain 4–39 and its two derived deletion strains was similar, while wild-type H4–8A formed less biomass than the Δ*sc3*Δ*sc15* strain (Figure 2B). Wild-type 4–39 produced 1.59 g L^–1^ extracellular material, of which 0.27 g L^–1^ schizophyllan (Figure 2C,D). By contrast, wild-type H4–8A produced 7.43 g L^–1^ extracellular material, of which 3.63 g L^–1^ schizophyllan. Inactivation of *sc3* and *sc3* and *sc15* in the 4–39 background significantly increased release of extracellular material (5.37 and 4.55 g L^–1^, respectively) including schizophyllan (1.83 and 2.62 g L^–1^, respectively). In fact, these levels were not significantly different from the wild-type H4–8A. Notably, sediment biomass remained similar between the Δ*sc3* and the Δ*sc3*Δ*sc15* and the parental 4–39 strains, suggesting that the additional extracellular polysaccharides were produced in greater amounts rather than simply being redistributed from existing biomass. This pattern is consistent with previous work showing that inactivation of *sc3* in *S. commune* increases schizophyllan production and alters hyphal wall composition, particularly by increasing the water-soluble glucan fraction.^15^ In addition, ATR-FTIR analysis has previously been performed on mycelium of wild-type strain 4–39 and its Δ*sc3* derivative and showed broadly similar bulk spectra despite these known differences in glucan distribution.^20^ It was proposed that the interaction of cell wall-located SC3 hydrophobin with schizophyllan prevents the release of this polysaccharide in the culture medium. Indeed, it was shown that SC3 can interact with schizophyllan and β-(1–3)-glucan.^17^ Our results show that inactivation of *sc15* in a Δ*sc3* background does not further increase the release of schizophyllan. So far, it is not clear what the composition of the other released molecules in the culture medium is.

**Figure 2:**
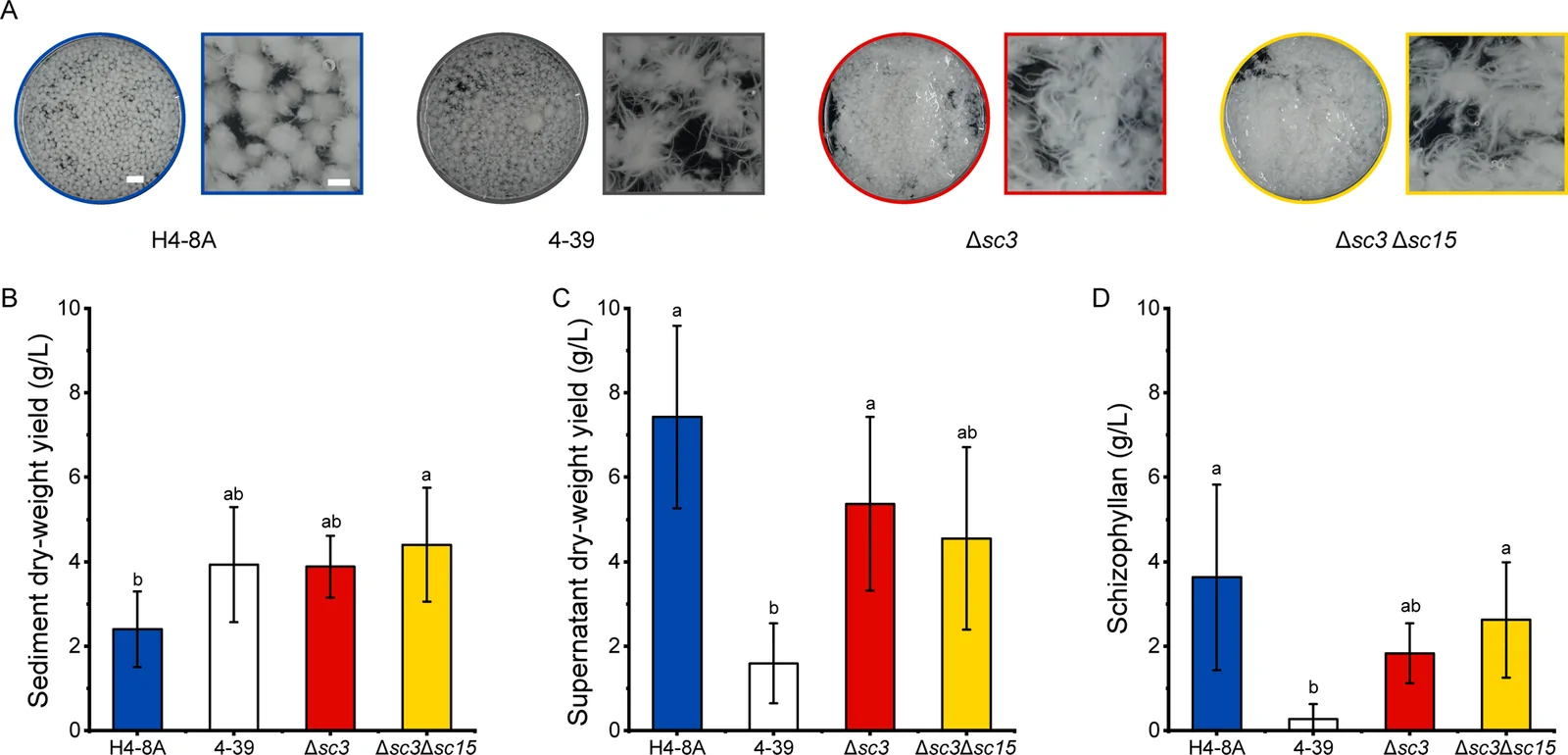
Pellet morphology and material distribution during liquid incubation. (A) Overview and close-up images of mycelial pellets. (B) Sediment dry-weight yield after centrifugation. (C) Supernatant dry-weight yield, defined as the dry weight of material recovered from the culture supernatant after centrifugation. (D) Schizophyllan yield recovered from the supernatant by ethanol precipitation. Different letters indicate significant differences among strains (*p* ≤ 0.05). Scale bars in (A): 10 mm (overview) and 2 mm (close-up).

### Static Incubation

The wild-type strains H4–8A and 4–39 and the Δ*sc3* and the Δ*sc3*Δ*sc15* strains were grown on wood, Teflon, and glass slides placed on agar medium. The wild-types showed similar growth behavior, fully covering the wood, Teflon, and glass with a dense white mycelium (Figure 3A). The Δ*sc3* and the Δ*sc3*Δ*sc15* strains also fully colonized the substrate surfaces. However, aerial hypha formation was reduced, in particular on glass and wood (Figure 3A). Scanning electron microscopy of Δ*sc3*Δ*sc15* grown on wood showed an uneven surface structure, with regions containing sparse aerial hyphae and regions dominated by exposed vegetative hyphae (Figure 3B–D).

**Figure 3:**
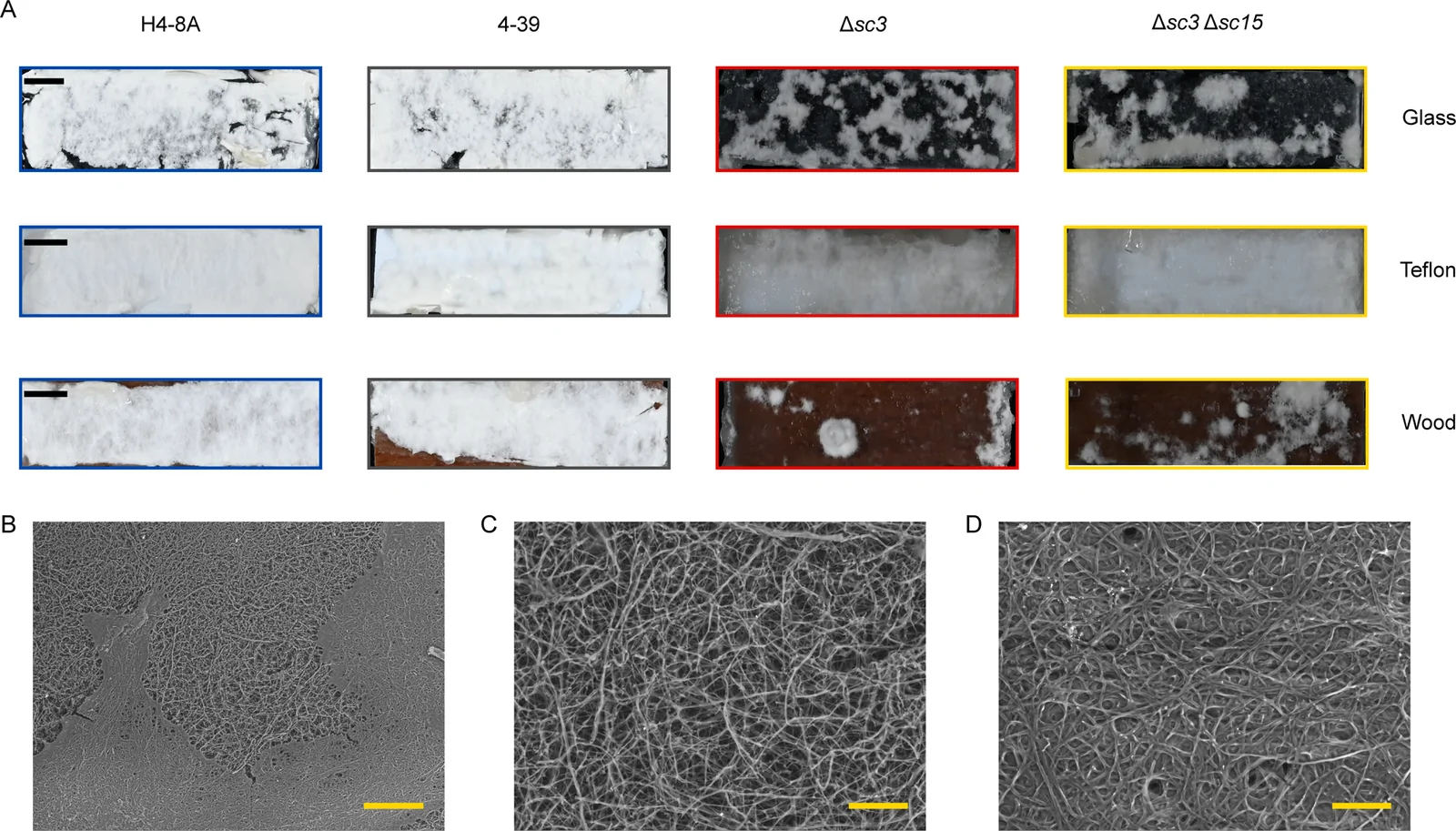
Surface morphology of mycelium grown statically on glass, wood, and Teflon. (A) Representative images of mycelial growth on glass, Teflon, and wood after static incubation. (B–D) SEM images of Δ*sc3Δsc15* mycelium grown on wood. Panel B shows an overview of the surface of the mycelium. Panels C and D show regions with sparse aerial hyphae and regions lacking aerial hyphae, respectively. Scale bars: 10 mm for the glass row (A), 5 mm for the Teflon and wood rows (A), and 200 μm (B) and 50 μm (C and D).

Contact angle analysis provided further insight into the air-exposed surfaces of the mycelia growing on wood (Figure 4). The regions of the air-exposed sides of the mycelium of the Δ*sc3* and the Δ*sc3*Δ*sc15* strains that lacked aerial hyphae showed mean contact angles of 65.7° and 54.4°, respectively. Regions with aerial hyphae of these strains were hydrophobic with average values of ∼96°, but the water was readily absorbed in the mycelium. The surfaces of the wild-type strains were super hydrophobic with contact angles >120°. These observations are consistent with previous reports showing that SC3 and SC15 contribute to aerial hyphae formation and surface hydrophobicity in *S. commune*.^7,10,12,21^

**Figure 4:**
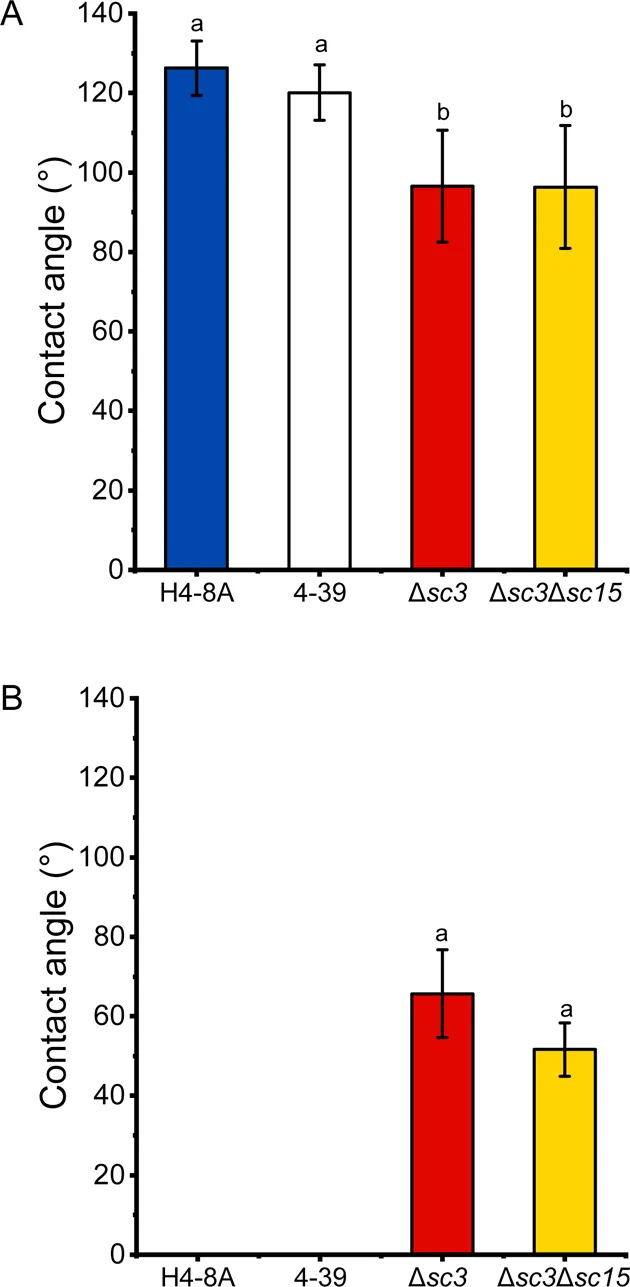
Contact angle of regions of the mycelium with (A) and without (B) aerial hyphae after growing under static conditions on wood. Different letters indicate significant differences among strains (*p* ≤ 0.05). No measurements were obtained for vegetative hyphae of wild-type strains because of complete aerial hyphal coverage.

Because attachment was expected to depend on substrate surface properties, the water contact angles of the uncolonized substrates were first measured. Glass, Teflon, and wood veneer showed distinct wettability, with contact angles of 15.16 ± 1.7°, 113.30 ± 2.8°, and 84.91 ± 4.1°, respectively. The attachment strength of mycelium of the wild-type strains and the Δ*sc3* and the Δ*sc3*Δ*sc15* strains after growing for 6 days on these surfaces was evaluated by a stepwise washing detachment assay. On wood, both wet mycelium (i.e., fresh cultures grown on the wood substrate and immediately tested for adhesion) and dried mycelium remained attached at the maximum tested speed of 350 rpm. Because all samples remained attached at 350 rpm, their detachment thresholds on wood exceed the tested range. This indicates strong physical entanglement or mechanical interlocking with the porous wood surface, irrespective of strain or hydration state. By contrast, fresh mycelium of all strains was easily removed from glass and Teflon, detaching during the initial 30 min cycle at 50 rpm. Dried mycelium grown on glass or Teflon showed higher adhesion. Most replicates grown on glass detached between 50 and 250 rpm, and no strain resisted detachment better than others (Figure 5). By contrast, dried wild-type mats adhered more strongly than the deletion strains on the hydrophobic Teflon. Wild-type strains H4–8A and 4–39 largely remained attached even at 350 rpm, whereas both deletion strains detached at lower speeds (50–250 rpm and 150–250 rpm, respectively) (Figure 5). These results are in line with previous work showing that SC3 and SC15 are involved in attachment to hydrophobic surfaces.^11,12^ Data also show a clear difference between man-made materials (glass and Teflon) and a natural material (wood). Wood is a natural substrate of *S. commune*,^22^ and its composition and surface topography differ from that of glass and Teflon. It would be interesting to assess how impactful the surface topography of wood is compared to its chemical composition.

**Figure 5:**
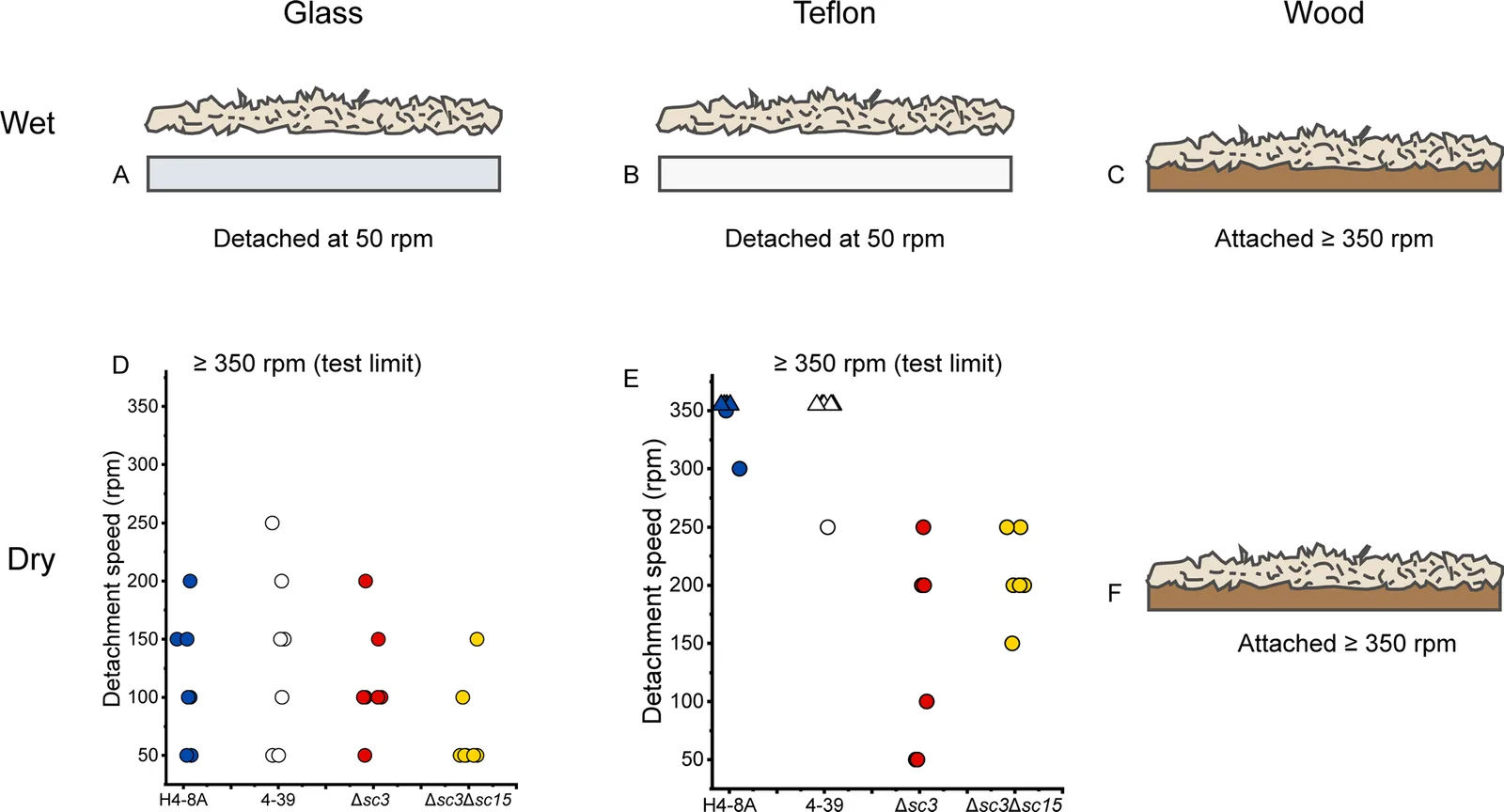
Surface attachment of mycelium on different substrates. Fresh (A–C) and dried (D–F) mycelium on glass (A,D), Teflon (B,E), and wood (C,F). Each point represents a replicate. Circles indicate detachment speed, and triangles indicate samples that remained attached at the maximum tested speed.

### Bonding Properties

Lap-shear tests were used to assess the adhesive performance of mycelium from liquid shaken and static substrate-grown conditions. To this end, fresh mycelium from liquid shaken culture was added between two birch veneer strips at a wet spread rate of 200 g m^–2^. For the static-incubation samples, two birch veneer pieces carrying directly grown surface mycelium were overlapped with the mycelial surfaces facing each other, following the surface mycelium bonding configuration used in previous veneer-bonding studies.^5,6^ All samples were pressed at 120 °C under 1 MPa of pressure.

Biomass from liquid shaken cultures of Δ*sc3* showed the highest bonding strength (1.83 MPa), exceeding that of Δ*sc3*Δ*sc15* (1.42 MPa) and 4–39 (1.45 MPa) but not H4–8A (1.53 MPa) (Figure 6A). Because the biomass fraction was applied at a fixed wet-based spread rate, differences in sediment solid content resulted in different dry biomass loadings at the bonding interface (Table 1). The higher bonding strength of Δ*sc3* cannot be explained simply by greater dry biomass loading. Although Δ*sc3* had the highest mean bonding strength, its calculated dry biomass loading was only intermediate among the strains, whereas 4–39 had the highest dry biomass loading but lower bonding strength (Table 1). This indicates that factors beyond dry biomass loading contributed to the observed bonding performance. Across the strains, the amount of schizophyllan recovered from the culture did not show a consistent relationship with lap-shear strength, indicating that bulk schizophyllan production alone was not predictive of bonding performance.

**Figure 6:**
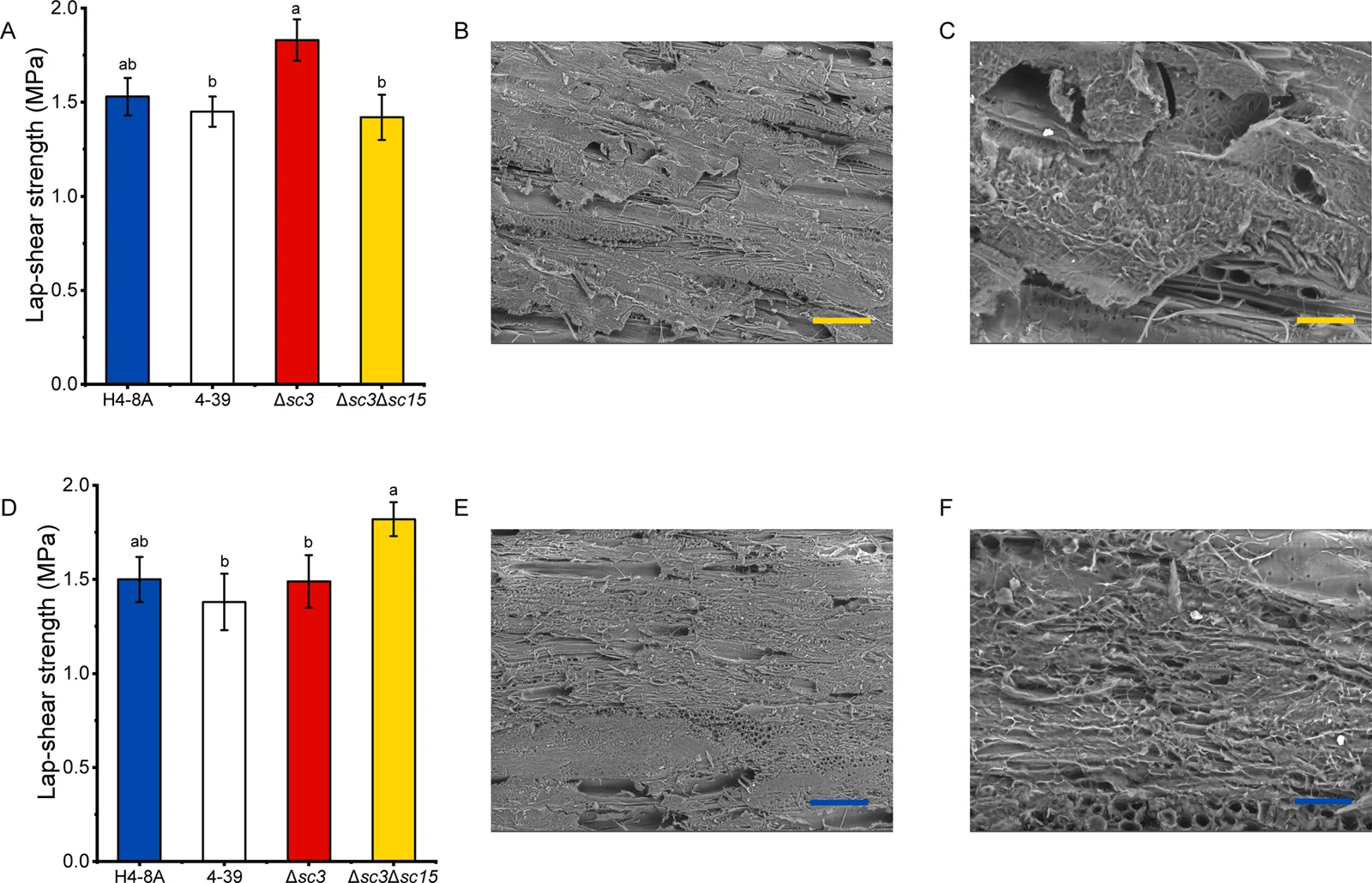
Lap-shear strength and representative SEM images of fractured bonded veneer surfaces. Lap-shear strength of the biomass of different strains grown in liquid shaken culture (A) and using static substrate-grown mycelium directly grown on veneer (D), and scanning electron microscopy of the resulting fractured surfaces from Δ*sc3*Δ*sc15* liquid shaken culture-derived bonds (B,C) and H4–8A static-incubation bonds (E,F). Different letters indicate significant differences among strains within each cultivation method (*p* ≤ 0.05). Scale bars: 200 μm (B,E); 50 μm (C,F).

**Table 1:** Sediment Solid Content and Calculated Dry Biomass Loading for Liquid-Culture-Derived Bonding Samples^a^

Strain	Sediment solid content (%)	Calculated dry biomass loading (g m^–2^)
H4–8A	1.47 ± 0.5	2.94 ± 1.0
4–39	4.80 ± 1.3	9.60 ± 2.7
Δ*sc3*	1.88 ± 0.2	3.76 ± 0.4
Δ*sc3Δsc15*	2.11 ± 0.7	4.22 ± 1.5

a Values are mean ± standard deviation. Dry biomass loading was calculated from the wet spread rate of 200 g m^–2^ and the measured sediment solid content.

In static incubation, Δ*sc3*Δ*sc15* showed the highest bonding strength (1.82 MPa), which was significantly higher than its parental wild-type strain 4–39 (1.31 MPa) and strain Δ*sc3* (1.38 MPa) but not significantly different from H4–8A (1.54 MPa) (Figure 6D). The Δ*sc3*Δ*sc15* strain showed regions with reduced aerial hyphae and greater exposure of vegetative hyphae; however, the present data do not establish a direct relationship between this surface morphology and the higher lap-shear strength.

Across both cultivation formats, the lap-shear strengths ranged from 1.31 to 1.83 MPa. These values are comparable with previously reported values for fungal mycelium-assisted wood bonding, including wild-type *Trametes versicolor* on yellow birch veneer^5,6^ and wild-type *Pycnoporus sanguineus* or *Trametes versicolor* on poplar veneer.^23^

Representative SEM images of fractured surfaces from both liquid shaken culture-derived bonds and static-incubation bonds showed residual fungal material on the opposing veneer surfaces (Figure 6B,C,E,F). This indicates that failure occurred mainly within the compressed fungal layer, rather than by clean separation at the wood–mycelium interface, supporting a cohesive-dominated failure mode. Taken together, the two bonding formats produced lap-shear strengths within a similar range. However, they represent different bonding configurations and were not normalized by the same dry adhesive loading.

## Conclusion

This study showed that deletion of *sc3* impacts extracellular polysaccharide secretion, surface hydrophobicity, and substrate-dependent adhesion in *S. commune*. Deletion of *sc15* in the Δ*sc3* background did not impact these phenotypes but it may have an effect on the lap-shear strength.

Deletion of *sc3* alone or together with *sc15* increased crude schizophyllan production relative to the 4–39 parental strain while maintaining comparable sediment biomass. Under static incubation, the deletion strains formed flatter and more hydrophilic mats with reduced aerial hyphae formation. Attachment and bonding depended strongly on substrate and growth conditions. All strains attached strongly to porous wood, consistent with mechanical interlocking. On hydrophobic Teflon, dried wild-type mats showed stronger retention than the deletion strains. By contrast, lap-shear bonding strength varied among strains, with the highest values obtained for Δ*sc3* and Δ*sc3*Δ*sc15* under liquid shaken and static cultivation conditions, respectively. Together, these findings show that changes in extracellular polysaccharide production, mycelial surface morphology, and wettability accompany genotype- and substrate-dependent differences in attachment and bonding, although the individual contributions of these factors cannot be resolved from the present data. Hydrophobin-related gene deletions therefore provide a potential route to tune fungal bonding behavior for biobased adhesive systems.
